# Switching to an alternative biological agent in juvenile idiopathic arthritis (II)

**DOI:** 10.1186/1546-0096-9-S1-P163

**Published:** 2011-09-14

**Authors:** S Murias, A Remesal, L Latorre, M Gomez, R Merino

## Background

Juvenile idiopathic arthritis (JIA) is a heterogeneous disease, and it’s associated with an increased use of various biological agents in recent years.

## Aim

Evaluate clinical response to current biological agent in JIA patients.

## Methods

This is a retrospective study of 109 JIA patients from a tertiary centre (see poster 1). Variables included were: current biological treatment (both the first agent used as the second, third or fourth when it had been switched) and duration of treatment (years with a new agent or since reintroduction of an agent used before) plus physician visual analogue scale (ph-VAS), erythrocyte sedimentation rate (ESR) and C-reactive protein (CRP) at baseline and at the end of the study. Concomitant therapy with methotrexate (MTX) was also recorded.

## Results

Duration of treatment with the current biological agent was 1.2 ± 1 (0.1- 4.9) median 0.8 years. This agent was etanercept (ETA) in 58 cases, adalimumab (ADA) in 16, tocilizumab (TCZ) in 11, anakinra (AK) in 9 and infliximab (IFX) in 2. Thirteen patients were off treatment with biological agents, 12 due to inactive disease and one because of inefficacy.

Concomitant treatment with MTX was observed in 8/58 (14%) patients with ETA, 4/16 (25%) with ADA, 5/11 (46%) with TCZ, 1/2 (50%) with IFX and none with AK. Table 1.

**Table 1:** Analytical and clinical response to current biological agent

	Ph-VAS				ESR				CRP			
	Initial*	End*	۵	p	Initial*	End*	۵	p	Initial*	End*	۵	p
	
ETA	3.25	0.5	-6	0.00	28	12	-5	0.00	13.2	2.3	-5	0.00
ADA	2.42	0.4	-3	0.01	17	13	-1	0.26	5.58	1.2	-2	0.06
AK	5.36	0.00	-3	0.01	62	9	-2	0.02	69.5	2.9	-2	0.03
TCZ	6.45	0.86	-3	0.01	46	4	-3	0.01	95.6	0.6	-3	0.01
IFX	4.9	0.00			47	6			32.8	0.9		

*Values are expressed as mean.

## Conclusion

The results indicate that reintroduction of a previously effective biological agent may be successful; besides, switching to a second, third or even fourth biological agent in those patients whose disease remains active, can be effective as well.

